# Overexpressed coiled-coil domain containing protein 8 (CCDC8) mediates newly synthesized HIV-1 Gag lysosomal degradation

**DOI:** 10.1038/s41598-020-68341-3

**Published:** 2020-07-10

**Authors:** Xiangxiang Jiang, Xiaopeng Jia, Jinhuan Sun, Chunxia Qi, Lingling Lu, Yanfeng Wang, Lei Zhang, Min Wei

**Affiliations:** 1 School of Medicine, Nankai University, Tianjin, China; 2 Nankai University Second People’s Hospital, School of Medicine, Nankai University, Tianjin, China

**Keywords:** Translational research, Virus-host interactions

## Abstract

Normally, HIV-1 enters into CD4+ cells through membrane fusion, and newly synthesized HIV-1 viral proteins assemble on the plasma membrane to form viral particles and bud out. In the previous study, we found host factor coiled-coil domain containing protein 8 (CCDC8) can strongly inhibit HIV-1 production, but the underline mechanism is not clear. Here we show that overexpression of CCDC8 reverses the normal HIV-1 production process, and causes newly assembled HIV-1 Gag particles to be endocytosed on the plasma membrane, rather than budding out. Live-cell imaging system captured the moment of CCDC8-mediated Gag internalization on the plasma membrane, and the speed of Gag turnover is up to 1.53 μm/s, much faster than Gag assembly on the plasma membrane. After Gag internalization, it accumulates in the cellular organelle—lysosome for degradation, but not proteasome, autophagosome, endoplasmic reticulum, clathrin or recycling endosome. In addition, CCDC8 is a membrane-associated protein, and N-terminal of CCDC8 is very important for membrane binding, and also important for inhibition of Gag assembly. C-terminal deletion of CCDC8 has a little effect on anti-HIV-1 effect. Moreover, CCDC8 is phosphorylated at amino acid threonine T87 and serine S261, and mono-methylated at lysine K491. Alanine mutations of T87A, S261A and K491A singly or in combination do not affect CCDC8 anti-HIV activity. In conclusion, overexpression of CCDC8 can cause newly assembled HIV-1 Gag particles on the plasma membrane to be endocytosed and degraded in lysosome.

## Introduction

Human Immunodeficiency Virus type 1 (HIV-1), the etiologic agent of AIDS, belongs to the retrovirus family^1, 2^. In its life cycle, HIV-1 first recognizes and then binds to the CD4+ receptor^2^. Under the aid of co-receptors, most importantly CCR5 or CXCR4, HIV-1 enters the target cells through membrane fusion^2^. HIV-1 uncoats its capsid and undergoes reverse transcription from genome RNA into double stranded DNA. Viral integrase then inserts the viral double stranded DNA into the human genome. The integrated viral DNA can be silent or be activated by viral accessary protein Tat^2^. Tat protein starts viral mRNA transcription in the nucleus and viral mRNAs export to the cytoplasm for translation. The translated viral proteins and transcribed viral genome RNA assemble on the plasma membrane, and then viral particles bud out and release^2^. The released mature viral particles infect new target cells and start a new life cycle. Human cells also encode proteins to facilitate or block the normal viral replication cycle. In the previous study, we reported that coiled-coil domain containing protein 8 (CCDC8) can inhibit HIV-1 Gag production^3^. We continue our study of CCDC8 against HIV-1 in this study.

Some important proteins include coiled-coil domains in the coding regions, for example anti-retrovirus factor Trim5α^4, 5^, Tetherin^6^, and etc. There are other coiled-coil domain containing proteins, temporarily as coiled-coil domain containing (CCDC) family proteins. However, functions of most family members remain unknown. Among them, CCDC3 is a secretory protein, expressed in vascular endothelial cells (ECs) and adipose tissue cells^7–10^. The CCDC3 homology on amino acid across species suggests the universal conserved functions. One study reported that CCDC3 represses TNF-α/NF-κB-induced pro-inflammatory response in ECs, which could be related to obesity and atherosclerosis^7^. Another study found that CCDC3 modulates liver lipid metabolism through interacting with TAp63, a member of p53 family^10^. These studies suggest that CCDC3 may play an important role in lipid metabolism. Another member CCDC109B was reported that it is highly expressed in gliomas, and silencing of CCDC109B attenuates glioma proliferation and migration^11^. CCDC109B could be related to cancer development.

The function of CCDC8 is still elusive. Mutations in CCDC8 cause a genetic disorder of 3-M syndrome, an autosomal recessive primordial dwarfism^12, 13^. 3-M syndrome patients present pre- and postnatal growth retardation, and bone abnormalities^13–15^. 3-M syndrome could be also related to the mutations of either Obsl1 (cytoskeleton protein obscuring-like 1) or E3 ligase Cul7 (Cullin 7)^12–16^. Immunoprecipitation tests prove the interaction between CCDC8 and Obsl1 and Cul7, and that they are in a common pathway^3, 14, 15^. One study identified Obsl1 as a human papillomavirus (HPV) capsid protein L2 interacting protein to facilitate HPV endocytosis^17^. CCDC8 was also reported to be associated with hepatitis B virus (HBV)-related hepatocellular carcinoma in Southern China^18^. But the underline mechanism is still unknown.

In the previous study, we reported that overexpression of CCDC8 in human cells inhibits HIV-1 production^3^. The decrease production of HIV-1 is relative to HIV-1 Gag assembly defect. CCDC8, a membrane associated protein, interacts with HIV-1 Gag and causes HIV-1 Gag polyubiquitination, internalization and degradation^3^. However, it is still elusive how CCDC8 prevents HIV-1 Gag assembly and release from cells. What is the function of CCDC8 in different species? Could we trace the CCDC8-mediated Gag internalization in real time? Where do internalized Gag proteins go after internalization? Which part of CCDC8 is important for inhibition of Gag assembly? In this study, we try to answer the questions, and further map the regions of CCDC8 to interact with HIV-1 Gag. We also trace the moving of CCDC8-mediated HIV-1 Gag internalization in live cell imaging system. Finally, we localize the CCDC8-mediated Gag particles in the cells and analyze posttranslational modifications of CCDC8.

## Materials and methods

### Cells and plasmids

HEK293T (CRL-11268, ATCC) cells were cultured in Dulbecco’s Modified Eagle Medium supplemented with 10% fetal bovine serum. Plasmid vGag/GagPol-RRE (GPV-RRE), a Rev-dependent vector expressing HIV-1 Gag and GagPol protein, was a gift from Dr. Chen Liang (McGill University, Canada). The truncated CCDC8 expression plasmids in N-terminal-histidine-tagged pTT5 vector were constructed with PCR and confirmed by sequencing. The primers are listed in Supplementary data (Table S1).

Fluorophore-tagged plasmid vGag-GFP was a gift from Dr. Andrew Mouland in McGill University, Canada (NIH AIDS Research Reagent Program, Catalog number 11468). vGag-GFP was replaced with blue fluorescence protein (BFP) tag by digestion with restriction endonuclease BamHI and XbaI. Because XbaI site is methylated by Dam in vGag-GFP plasmid produced from competent cells DH5α, XbaI endonuclease can not digest vGag-GFP. Thus, we used dam^-^ Trans110 competent cells to transform the plasmid and successfully completed BamHI and XbaI digestion of vGag-GFP.

Cellular organelle markers were all acquired from a nonprofit company Addgene (https://www.addgene.org), including pEGFP-LC3 (autophagosome marker, catalog No. 24920, a gift from Toren Finkel^19^), Clathrin-LCa-EYFP (clathrin, catalog No. 21741, a gift from Xiaowei Zhuang^20^), mCherry-Rab5 (early endosome marker, catalog No.49201, a gift from Gia Voeltz^21^), Rtn4a-GFP (endoplasmic reticulum marker, catalog No. 61807, a gift from Gia Voeltz^22^), mCherry-Rab7A (late endosome marker, catalog No. 61804, a gift from Gia Voeltz^23^), Lamp1-YFP (lysosome marker, catalog No. 1816, a gift from Walther Mothes^24^), DsRed-rab11 (recycling endosome marker, catalog No. 12679, a gift from Richard Pagano^25^), and Flag-HA-PSMD14 (26S proteasome marker, catalog No. 22557, a gift from Wade Harper^26^). Among them, red marker mCherry-Rab7A and DsRed-rab11 were reconstructed with green GFP tag. And GFP tag was also added to plasmid Flag-HA-PSMD14 (Table 1).

**Table 1:** The intracellular organelle marker in this study.

Marker name	Intracellular apparatus position of the markers
Lamp1-YFP	Lysosome
Clathrin-LCa-EYFP	Clathrin-mediated endocytosis
Rab5-GFP	Early endosome
Rab7A-GFP	Late endosome
EGFP-LC3	Autophagosome
Rab11-GFP	Recycling endosome
Rnt4a-GFP	Tubular ER
PSMD14-GFP	Proteasome

We cloned CCDC8 gene from HEK293T cells, and the sequence of CCDC8 was deposited into Genebank as accession number KT_894208^3^.

### Confocal microscopy

HEK293T cells were transfected (Lipofectamine 3000, Invitrogen) with indicated plasmids and cultured for 24 h, fixed in 4% paraformaldehyde for 5 min and followed by cold methanol at − 20 °C for 15 min. The cells stained with DAPI and examined under an Olympus FV1000 confocal microscope.

### Virus purification

Virus purification was described as previously^27, 28^. Briefly, HIV-1 viral like particle (VLP) expressing plasmid GPV-RRE and Rev (5:1) with or without truncated CCDC8 were cotransfected in HEK293T cells (Lipofectamine 3000, Invitrogen). After 48-h transfection, the VLP containing supernatant was passed through 0.22 µm filter, and centrifuged at 35,000 rpm for 1 h through 20% sucrose cushion in Beckman ultracentrifuge. The viral pellets and cell lysates were analyzed by Western blot.

### Live-cell imaging

HEK293T cells were transfected with vGag-GFP and mCherry-CCDC8 (molar ratio 1:2) or empty vector. Tracking Gag in live cells was performed at 5% carbon dioxide and controlled humidity and temperature at 37 °C on an Olympus IX81 microscope. Software Imaris and Image J were used to analyze movies and images.

### Immunoprecipitation

The immunoprecipitation was performed as previously described^3^. Briefly, HEK293T cell lysates, expressing Flag-CCDC8, were incubated with anti-Flag M2 affinity beads (Catalog No. A2220, Sigma-Aldrich) at 4 °C overnight. After washing with the buffer, the beads were eluted with 3 × flag peptide (Catalog No. F4799, Sigma-Aldrich), and then resolved by SDS-PAGE and stained with Coomassie blue or analyzed by Western blot.

### Mass spectrometry

CCDC8 protein was purified by above immunoprecipitation, and in the SDS-PAGE gel the CCDC8 band was cut and analyzed by nanoliquid chromatography/tandem mass spectrometry (nano-LC–MS/MS) in BTM Biolabs (Hanzhou, China).

## Results

### Phylogenetic analysis of CCDC8 genes

**Figure 1:**
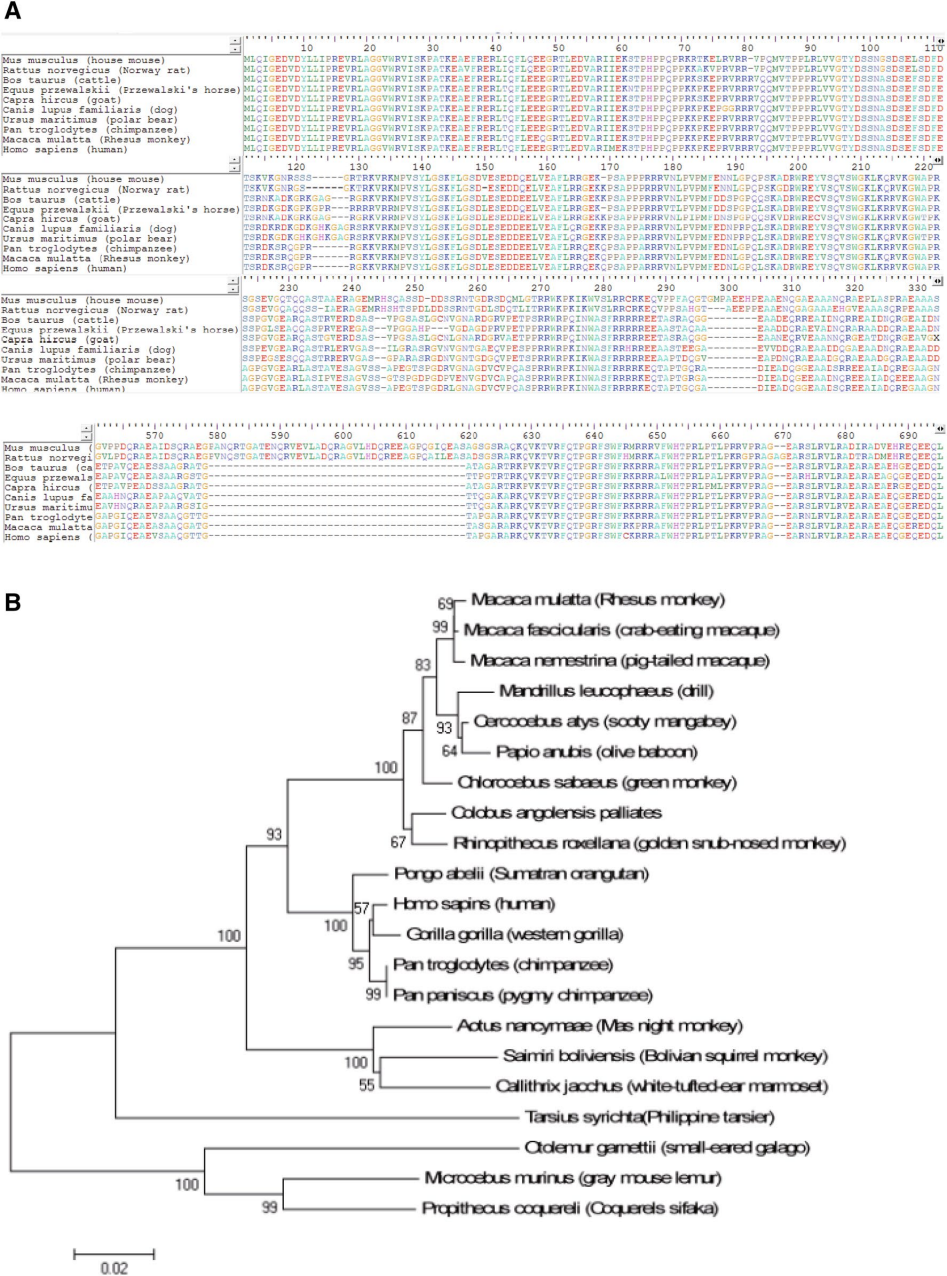
Comparison of CCDC8 amino acids across species and phylogenetic analysis of primates’ CCDC8 genes. (**A**) Alignment of CCDC8 amino acid sequences across species. (**B**) Phylogenetic analysis of primates’ CCDC8 genes. Phylogenetic tree was constructed by Neighbor-Joining method, and bootstrap values were showed in the branch.

First, we downloaded the sequences of CCDC8 genes and proteins from Genebank (https://www.ncbi.nlm.nih.gov). The CCDC8 protein sequences were edited and aligned by software Bioedit (https://bioedit.software.informer.com), and showed in Fig. 1A. CCDC8 amino acids are rather conservative at N and C terminals in mice, rats, cattle, horses, goats, dogs, monkeys and humans (Figs. 1A, S1). We also compared the CCDC8 amino acids in primates (Fig. S2). The N-terminal first 220 amino acids of CCDC8 protein were almost same in different primates, and approximately 100 amino acid sequences at the C terminal are extraordinarily similar (Fig. S2). CCDC8 homology suggests that it could play a very important role in normal physiology. Moreover, the Genebank primates' CCDC8 genes were analyzed using Neighbor-Joining (NJ) method, and the evolutionary tree was shown in Fig. 1B. In this evolutionary tree, CCDC8 gene of human is more close to ones of gorilla (Gorilla) and chimpanzee (Chimpanzee) than others’. The Bootstrap value is as high as 95% with the statistical significance. But the human CCDC8 gene is slightly distant from other primates, such as *Rhesus monkey* and *Cercocebus atys*. The relationship between CCDC8 in primates is similar to our evolutionary relationship in primates.

### Membrane localization signal of CCDC8 protein

**Figure 2:**
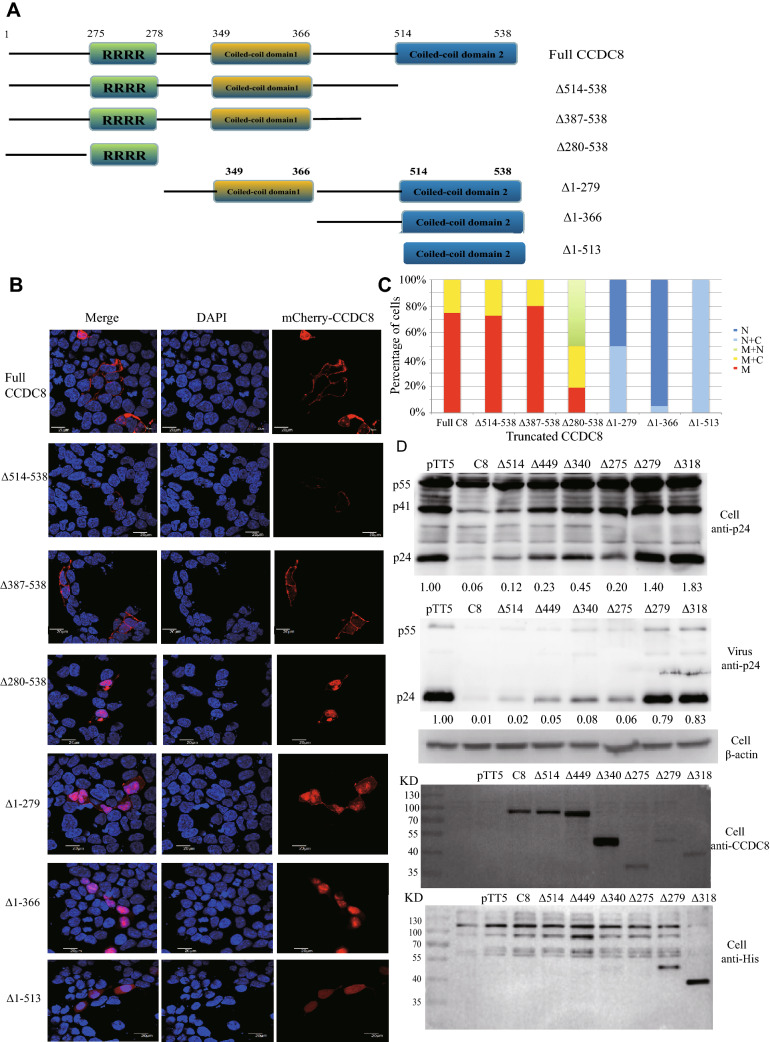
Identification of CCDC8 membrane localization signal and fine mapping of CCDC8 region against HIV-1 production. (**A**) Schematic diagram of CCDC8 truncation. (**B**) Representative immunofluorescence images of expression of truncated CCDC8 proteins. The HEK293T cells were fixed and stained by DAPI. The scale bar is 20 µm. (**C**) Quantitative analysis of truncated CCDC8 localization. Around 50 to 100 cells were calculated. M, membrane only; M + C, membrane and cytoplasm; M + N, membrane and nucleus; N + C, nucleus and cytoplasm; N, nucleus only. (**D**) Western blot analysis of effect of truncated CCDC8 protein on the HIV-1 production. Viral GPV-RRE and Rev were cotransfected with pTT5 vector or CCDC8 (C8), or ∆514–538 (∆514), or ∆449–538 (∆449), or ∆340–538 (∆340), or ∆275–538 (∆275), or ∆1–279 (∆279), or ∆1–318 (∆318). The experiments were repeated three times, and the typical blot was shown. The values under the blot pictures stand for the relative p24 intensity compared to the cotransfection of GPV-RRE and pTT5 vector.

In our previous study, CCDC8 protein was confirmed as a membrane-associated protein^3^. However, the membrane localization signal is unknown. To explore that, we truncated CCDC8 proteins from N- and C-terminal respectively, with or without red fluorophore mCherry tag (Fig. 2A). Because the structure of CCDC8 is unresolved, the diagram of CCDC8 motifs with two coiled-coil domains and one arginine-rich region shown in Fig. 2A, is only based on published bioinformatics analysis and an article^29^. The full sequence of CCDC8 contains 538 amino acids (Genebank, NM_032040), but our CCDC8 protein, which was cloned from HEK293T cells, contains 519 amino acids (Genebank, KT_894208) due to the premature stop codon in the C terminal. When C terminal mCherry- tagged CCDC8 was constructed, the stop codon was deleted and mutated back to 538 amino acids.

Different truncated CCDC8 constructs with mCherry tag were transfected into HEK293T cells. After culture, the cells were fixed and examined under a confocal microscope (FV1000, Olympus). The immunofluorescence results are shown in Fig. 2B.

Although CCDC8 is a membrane- associated protein and localizes on the plasma membrane, the clumps of CCDC8 proteins do appear in the cytoplasm in some cells (~ 25% cells) (Fig. 2B). Immunofluorescence data showed that when coiled-coil domain 2 was deleted (Δ514-538), the localization of truncated CCDC8 in cells was not affected compared to the full CCDC8 (Fig. 2B,C). Based on the cell counting, in ~ 73% HEK293T cells, the truncated CCDC8 protein (Δ514-538) localizes on the cell membrane only, while in the remaining 27% cells it is on the cell membrane and aggregates in the cytoplasm simultaneously. Similarly, deletion of Δ387-538 in CCDC8 does not affect the location on the cell membrane. In ~ 80.4% cells, Δ387-538 CCDC8 localizes on the cell membrane only, while in 19.6% cells, it appears on the cell membrane and aggregates in the cytoplasm. However, when the C terminal of 280–538 amino acids were deleted (Δ280-538), only a small proportion cells ~ 19% shows the membrane localization; in most of cells, Δ280-538 CCDC8 appears in the cytoplasm or the nucleus or both, which does not occur in the other C-terminal truncated CCDC8 (Fig. 2B,C).

On the contrary, N-terminal deletions of CCDC8, Δ1–279, Δ1–366, Δ1–513, almost abandon the CCDC8 membrane localization property (Fig. 2B,C). In some cells, Δ1–279 CCDC8 protein accumulates in the nucleus and cytoplasm (Fig. 2B,C). Compared to the C terminal truncated CCDC8, Δ1–279 CCDC8 protein completely lost the property on the cell membrane only. While in ~ 95% cells, Δ1–366 CCDC8 concentrates in the nucleus (Fig. 2B,C). Together, these data show that N-terminal of CCDC8 is very important for membrane localization, and membrane localization signal could fall between 1 to 366 amino acids of CCDC8.

### Fine mapping of CCDC8 for inhibition of HIV-1 production

Next, we tried to confirm which truncated CCDC8 protein has the ability to inhibit HIV-1 production. Figure 2D shows the Western blot data of truncated CCDC8 expression and anti-HIV-1 activity. When we constructed the truncated CCDC8 without mCherry tag, the result constructs were not exactly as mCherry-tagged truncated CCDC8, since there are many repeated nucleic acid sequence in CCDC8. The C-terminal truncated CCDC8 proteins, Δ514–538 (Δ514), Δ449–538 (Δ449), Δ340–538 (Δ340), Δ275–538 (Δ275) still have anti-HIV-1 production ability, although diminished a little (Fig. 2D). In contrast, N-terminal truncated CCDC8 proteins, Δ1–279 (Δ 279), Δ1–318 (Δ 318), completely lost anti-HIV-1 ability (Fig. 2D). N-terminal of CCDC8 is also the membrane localization signal. These data agree with the previous conclusion that HIV-1 Gag first binds CCDC8 on the plasma membrane. The expression of Δ1–279 and Δ1–318 CCDC8 seemed to be low in cells when anti-CCDC8 polyclonal antibody was used, but it was not low when anti-His tag antibody was used (Fig. 2D). This could be explained by the antibody preference. The problem of this anti-His antibody is too much non-specific bands (Fig. 2D, lower panel). These data indicate that N-terminal of CCDC8 is very important in anti-HIV production.

### Live cell imaging system

**Figure 3:**
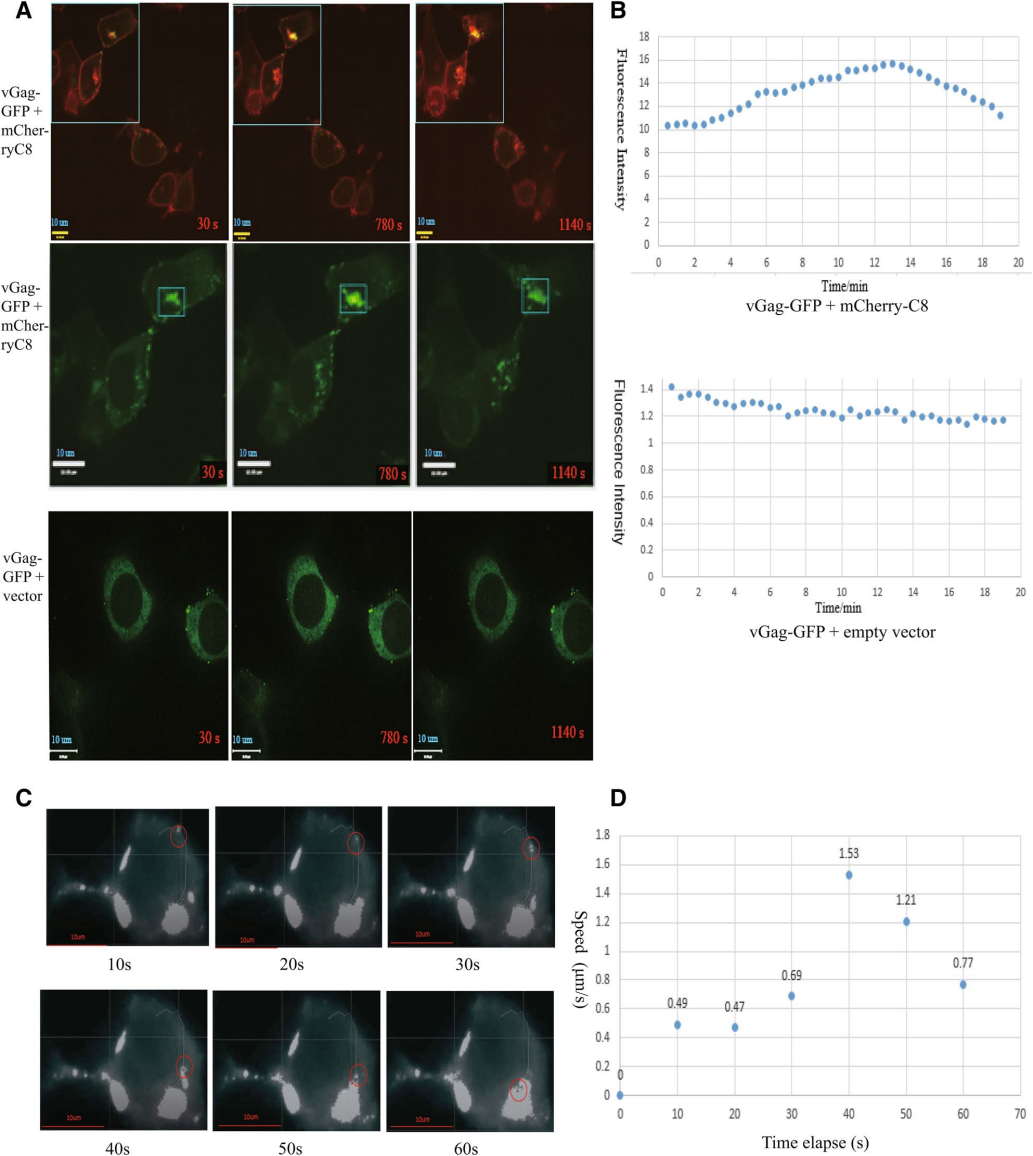
Live-cell imaging analysis of CCDC8-mediated HIV-1 Gag endocytosis at 24 h post transfection. (**A**) Immunofluorescence intensity tracing in live HEK293T cells in the cotransfection of vGag-GFP with mCherry-C8 group (upper panel) and vGag-GFP with empty vector group (lower panel). The red word in the right bottom corner stands for the time elapse. The scale bar is 10 µm. (**B**) Plot of normalized GFP intensity both in the experimental group and the control group in (**A**). (**C**) A movie of CCDC8-mediated HIV-1 Gag endocytosis. The red circle indicates the moving Gag particles. The scale bar is 10 µm. (**D**) Plot of speed of CCDC8-mediated Gag endocytosis.

In our previous study, immunofluorescence was carried out only in fixed cells. However, live cell imaging system can show HIV-1 Gag movement in cells at the physiological conditions, making the data closer to the truth. HEK293T cells were transfected with vGag-GFP and empty vector or mCherry-CCDC8, respectively. After 24 h, the live cells were observed. The images were analyzed by software Imaris and Image J. Considering the cytotoxicity of laser to cells, the time interval was set to 30 s and the images were acquired within 30 min. When vGag-GFP was co-transfected with mCherry-CCDC8, some intracellular bright vGag-GFP green puncta can be seen (Fig. 3A). The green fluorescence normalized intensity data was plotted in Fig. 3B. The intensity of these intracellular green puncta shows from weak to strong gradually, and then weak again (Fig. 3A middle panel, 3B upper panel). This phenomenon only occurred in the vGag-GFP and mcherry-CCDC8 co-transfected group, but not in the vGag-GFP and empty vector control group. The weak- strong- weak intensity of intracellular Gag suggests that Gag accumulates in the intracellular site first, and then degrades gradually. This phenomenon was not caused by fluorescence quenching due to the laser, because it did not appear in the control group.

Furthermore, we traced the newly synthesized Gag in real-time (Fig. 3C, S3 movie). According to the published articles^30^, Gag assembly is very fast. Thus, we changed the capture interval time to 10 s. A typical vGag-GFP punctum was captured (Fig. 3C, red circle), and it moved very fast, especially after arrival to the plasma membrane. The velocity of this puncta was recorded and plotted in Fig. 3D. At the beginning, the Gag punctum moved slowly and closely to the plasma membrane at velocity ~ 0.49 μm/s, and later it ran so fast at velocity 1.53 μm/s (Fig. 3C, 3D, between 30 and 40 s). At the time point 30 s, the Gag puncta was close to the plasma membrane, but it was inside of the cell at 40 s. This live cell imaging data clearly shows the CCDC8-mediated Gag endocytosis.

### Localization of CCDC8-mediated Gag endocytosis

**Figure 4:**
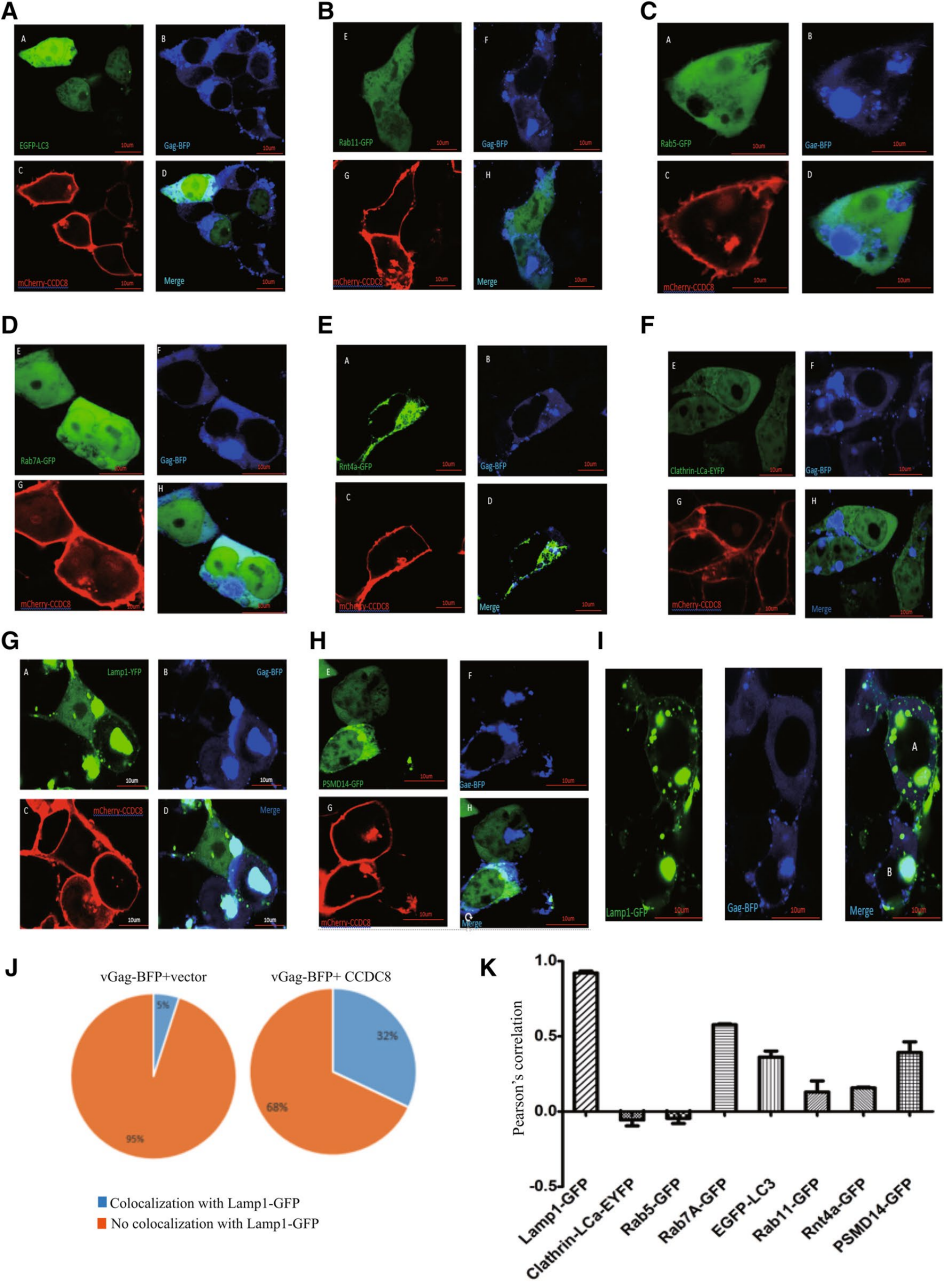
Localization of CCDC8-mediated internalized HIV-1 Gag in cellular organelles. Representative images are showed. (**A**) Autophagosome marker EGFP-LC3 and vGag-BFP and mCherry-CCDC8. (**B**) Recycling endosome marker Rab11-GFP and vGag-BFP and mCherry-CCDC8. (**C**) Early endosome marker Rab5-GFP and vGag-BFP and mCherry-CCDC8. (**D**) Late endosome marker Rab7A-GFP and vGag-BFP and mCherry-CCDC8. (**E**) Tubular endoplasmic reticulum marker Rnt4a-GFP and vGag-BFP and mCherry-CCDC8. (**F**) Clathrin marker and vGag-BFP and mCherry-CCDC8. (**G**) Lysosome marker Lamp1-YFP and vGag-BFP and mCherry-CCDC8. (**H**) Proteasome marker PSMD14-GFP and vGag-BFP and mCherry-CCDC8. (**I**) Lysosome marker Lamp1-YFP and vGag-BFP and empty vector. (**J**) Quantitative analysis of colocalization of vGag-BFP with Lamp1-GFP in the cotransfection of vGag-BFP with CCDC8 group or with empty vector group. P < 0.001. (**K**) Pearson’s correlation analysis of colocalization. Error bars represents standard deviation of results from dozens of cells.

We tried to know where Gag went after it was internalized by CCDC8, so we got and constructed some cellular organelle markers (Table 1), such as lysosome, early endosome, late endosome, autophagosome, tubular endoplasmic reticulum, and proteasome markers. Since the markers were labelled in green color, we replaced GFP tag of vGag-GFP with BFP (vGag-BFP, blue color). Thus, vGag-BFP and mCherry-CCDC8 and organelle marker were cotransfected into HEK293T cells. Figure 4 shows the co-localization data, surprisingly, only lysosome marker, Lamp1-YFP, co-localizes with CCDC8-mediated Gag puncta (Pearson’s colocalization coefficient 0.92, Fig. 4G), but other markers do not or partially (Fig. 4A–H,K, Pearson’s colocalization coefficient < 0.8). Interestingly, the Gag puncta are reversely correlated with clathrin marker and early endosome marker Rab5-GFP (Fig. 4C,F,K). In the control group, without mCherry-CCDC8, only vGag-BFP and Lamp1-GFP and empty vector contransfection shows that Gag puncta colocalize with Lamp1 only in 5% cells. In contrast, colocalization goes up to 32% cells in the group with mCherry-CCDC8 (Fig. 4I,J).

### Posttranslational modifications of CCDC8

**Figure 5:**
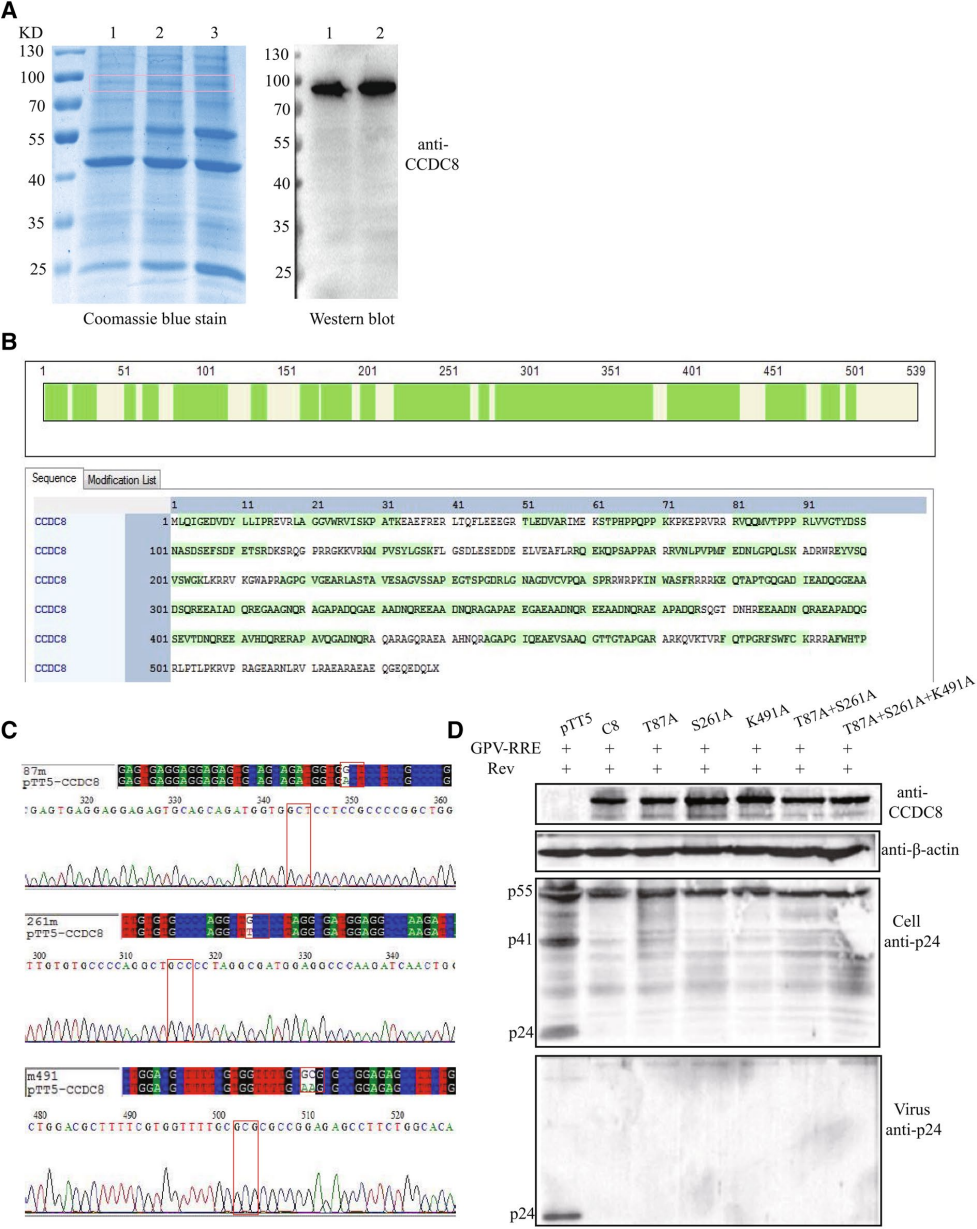
Effect of posttranslational modifications of CCDC8 against HIV-1. (**A**) Coomassie blue stain and Western blot analysis of purified CCDC8 protein by immunoprecipitation. The number 1, 2, 3 represents three times of repeats. The gel in frame was cut for LC–MS/MS analysis. (**B**) LC–MS/MS analysis shows the CCDC8 amino acid coverage in green color. (**C**) Mutations of T87A, S261A, and K491A were confirmed by sequencing, as shown in the chromatograph. (**D**) Western blot analysis of above mutations against HIV-1. The experiments were repeated for three times (n = 3), and the typical blot was shown.

To confirm the posttranslational modifications of CCDC8, we purified CCDC8 protein through immunoprecipitation and analyzed using mass spectrometry (Fig. 5A,B). Mass spectrometry identified 69.2% peptide of CCDC8 (Fig. 5B), indicating that the purified protein was CCDC8. We then examined the posttranslational modifications of CCDC8, including acetylation, phosphorylation, succinylation, crotonylation, propionylation, malonylation, glutarylation, ubiquitination, butyrylation, dihydroxyisobutyrylation, trihydroxybutyrylation, lysine methylation (monomethylation, dimethylation, trimethylation), arginine methylation (monomethylation, dimethylation). Only T87 and S261 in CCDC8 protein were found to be phosphorylated, and K491 was monomethylated (Table 2). Then, alanine mutation of CCDC8 T87A, S261A and K491A was performed singly or simultaneously (Fig. 5C). The results showed that single, dual or triple mutations of CCDC8 did not affect the anti-HIV effect (Fig. 5D).

**Table 2:** Posttranslational modification of CCDC8.

Name of sample	Type of modification	Peptides	Positions of modification
CCDC8	Phosphorylation	VQQmVt(ph)PPPR	87
CCDC8	Phosphorylation	LGNAGDVcVPQAs(ph)PR	261
CCDC8	Methylation	FSWFCK(methyl)	491

## Discussion

In the last study, CCDC8 and the pathway CCDC8-Obsl1-Cul7, were identified to inhibit HIV-1 production^3^. In this study, we provide more data to understand the CCDC8 gene and protein functions.

The N-terminal and C-terminal of CCDC8 proteins are rather conservative across species, suggesting its important functions. The coiled-coil 1 domain and coiled-coil 2 domain prediction came from a published article^29^. However, coiled-coil 2 domain deletion does not affect CCDC8’s membrane binding and anti-HIV activity (Fig. 2). Moreover, the author Nie et al. in another article predicts only one coiled-coil domain and some motifs of CCDC8^31^. Our data more agree with Nie’s domain analysis. We found many repeat nucleic acid sequences in CCDC8, resulting in the non-target constructs of truncated CCDC8 in PCR cloning. Despite that, we mapped the CCDC8 membrane localization signal on the N-terminal of CCDC8, which is also the region for inhibiting HIV-1 Gag assembly. N-terminal deletion results in the complete loss of anti-HIV-1 activity. These data are in accordance with the previous conclusion that HIV-1 Gag first interacts with membrane-associated CCDC8 protein, and then is internalized for degradation^3^.

In the last study, we only observed the internalization of Gag in fixed cells^3^. Here we show the movie of Gag moving in live cells at 5% CO_2_ and 37 °C, close to the physiology conditions. The movie clearly shows the CCDC8-mediated Gag endocytosis. The turnover velocity of Gag is 1.53 μm/s, really faster than its assembly. It is not clear why Gag reaches to the membrane and rebounds back quickly. CCDC8–Obsl1–Cul7 complex can induce Gag polyubiquitination^3^. Polyubiquitination could be the triggering signal for Gag endocytosis. Cargo proteins are usually endocytosed through clathrin-dependent or ubiquitin-dependent pathway. This data suggests the ubiquitin-dependent CCDC8-mediated Gag endocytosis, rather than clathrin-dependent pathway. This hypothesis was further supported by the cellular organelle localization data, which internalized Gag reversely correlated to the clathrin marker Clathrin-LCa-EYFP (Fig. 4F,K). The reverse correlation between CCDC8-mediated internalized Gag proteins and the clathrin marker Clathrin-LCa-EYFP and early endosome marker Rab5-GFP, almost excludes the possibility of Gag clathrin-dependent endocytosis. It seems that CCDC8’ mediated Gag directly goes into lysosome for degradation. We can not exclude that the internalized Gag first goes into late endosome, and then to lysosome, because the Pearson’s colocalization coefficient for late endosome marker Rab7A-GFP is 0.6, although less significant.

Previously, we thought polyubiqitinated Gag could go into proteasome for degradation^3^. However, the data here denied this speculation. Actually, the lysosome marker Lamp1-YFP provides more solid and convincing data. Furthermore, Fig. 3 shows that phenomenon of CCDC8-mediated Gag GFP weak–strong–weak intensity, supporting the Gag aggregate and degradation. The Gag degradation was proved previously by radioisotope ^35^S-labled methionine and cysteine pulse chase experiment^3^.

Most viruses, such as influenza virus, infect cells typically through clathrin-dependent endocytosis pathway^20^. Influenza virus then fuses with early and late endosome membrane for viral uncoating^32^. While, HIV-1 infects cells through membrane fusion, instead of clathrin-dependent endocytosis^2^. Viral entry belongs to exogenous viral antigen invasion. However, here we show the CCDC8-mediated newly synthesized viral Gag protein endocytosis. These Gag proteins belong to the endogenous viral proteins. Endogenous viral protein lysosome degradation always leads to antigen presentation. It is worth to investigate whether CCDC8-mediated Gag lysosome degradation is related to major histocompatibility complex (MHC) restrictive antigen processing and presentation.

The limitation of this study is that the data and conclusions are based on the overexpression of CCDC8. The biological function of CCDC8 is not clear. We also noticed that expression of CCDC8 in CD4^+^ T cells is very low. Because CCDC8 is a strong inhibitor of HIV-1 Gag assembly, low or no expression of CCDC8 explains successful replication of HIV-1 in these cells.

Posttranslational modifications of CCDC8 were identified as phosphorylation at amino acid T87 and S261, and mono-methylated at K491. Although these modifications were not found to affect CCDC8-anti-HIV activity, they could have other important functions for CCDC8.

In summary, this study provides more data to depict the function of CCDC8. Actually, in this and last study, we found a new pathway, in which newly synthesized HIV-1 Gag particles first assemble on the plasma membrane, then are endocytosed and degraded in lysosome. This pathway has the significance in anti-HIV drug development.

## Supplementary information

Supplementary file1 (MOV 111 kb)

Supplementary file2 (PDF 2680 kb)
