# Supplementary material for: Overexpressed coiled-coil domain containing protein 8 (CCDC8) mediates newly synthesized HIV-1 Gag lysosomal degradation
Source: Sci Rep. 2020 Jul 10;10:11416. doi: 10.1038/s41598-020-68341-3 (PMC7351720; doi:10.1038/s41598-020-68341-3)
Supplement: Supplementary file 2 — Supplementary file2 (PDF 2680 kb) [file 41598_2020_68341_MOESM2_ESM.pdf]

Supplementary Information

Overexpressed coiled-coil domain containing protein 8 (CCDC8) mediates newly synthesized HIV-1 Gag lysosomal degradation

Xiangxiang Jiang[¶1], Xiaopeng Jia[¶1], Jinhuan Sun[1], Chunxia Qi[1], Lingling Lu[1], Yanfeng Wang[1], Lei Zhang[1], Min Wei[1,2*]

1, School of Medicine, Nankai University, Tianjin, China

2, Nankai University Second People's Hospital, School of Medicine, Nankai University, Tianjin, China

¶ These authors contribute equally to this work.

Correspondence author: Professor Min WEI, weimin@nankai.edu.cn

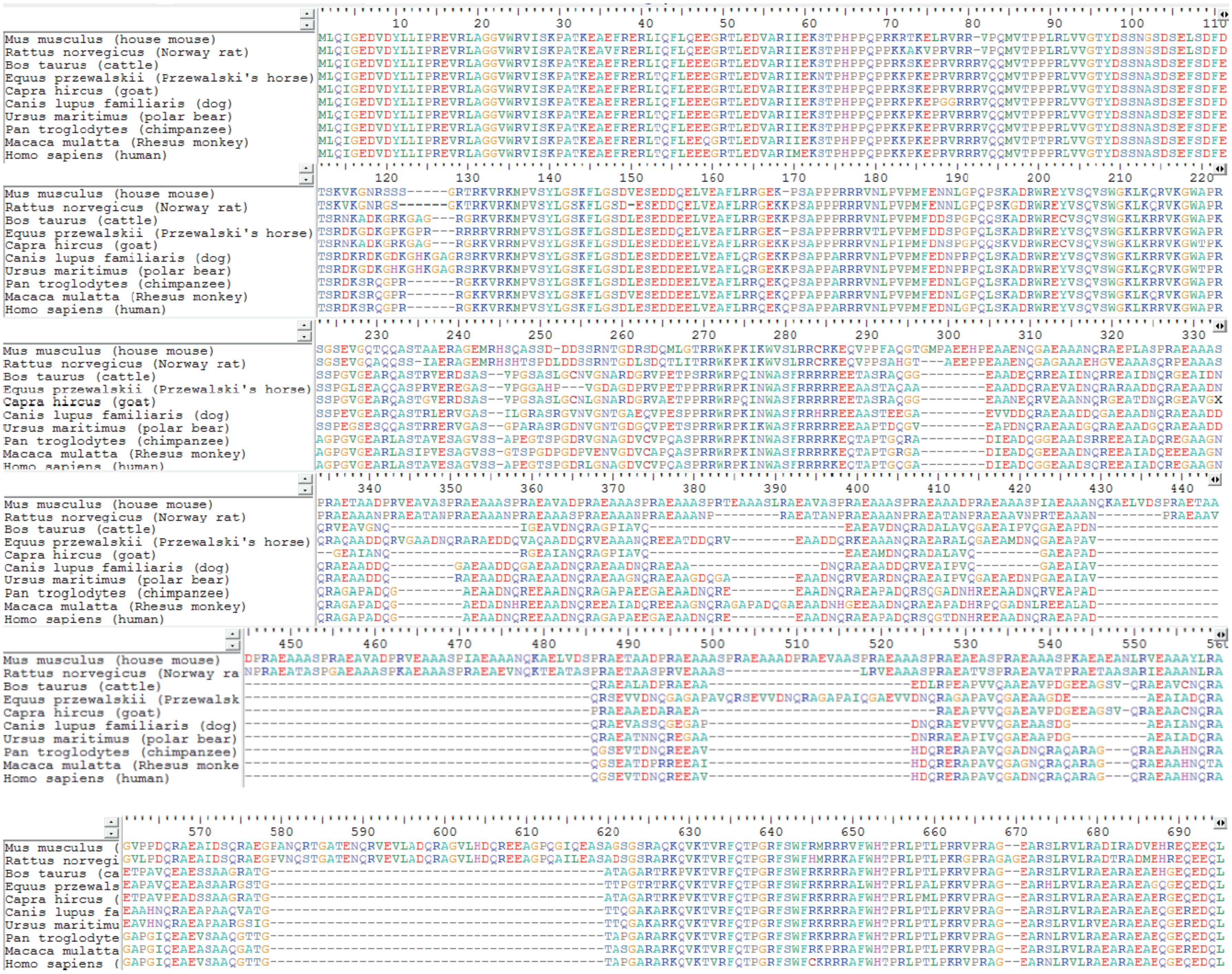

Supplementary Figure S1. Full amino acid sequences of CCDC8 across species.

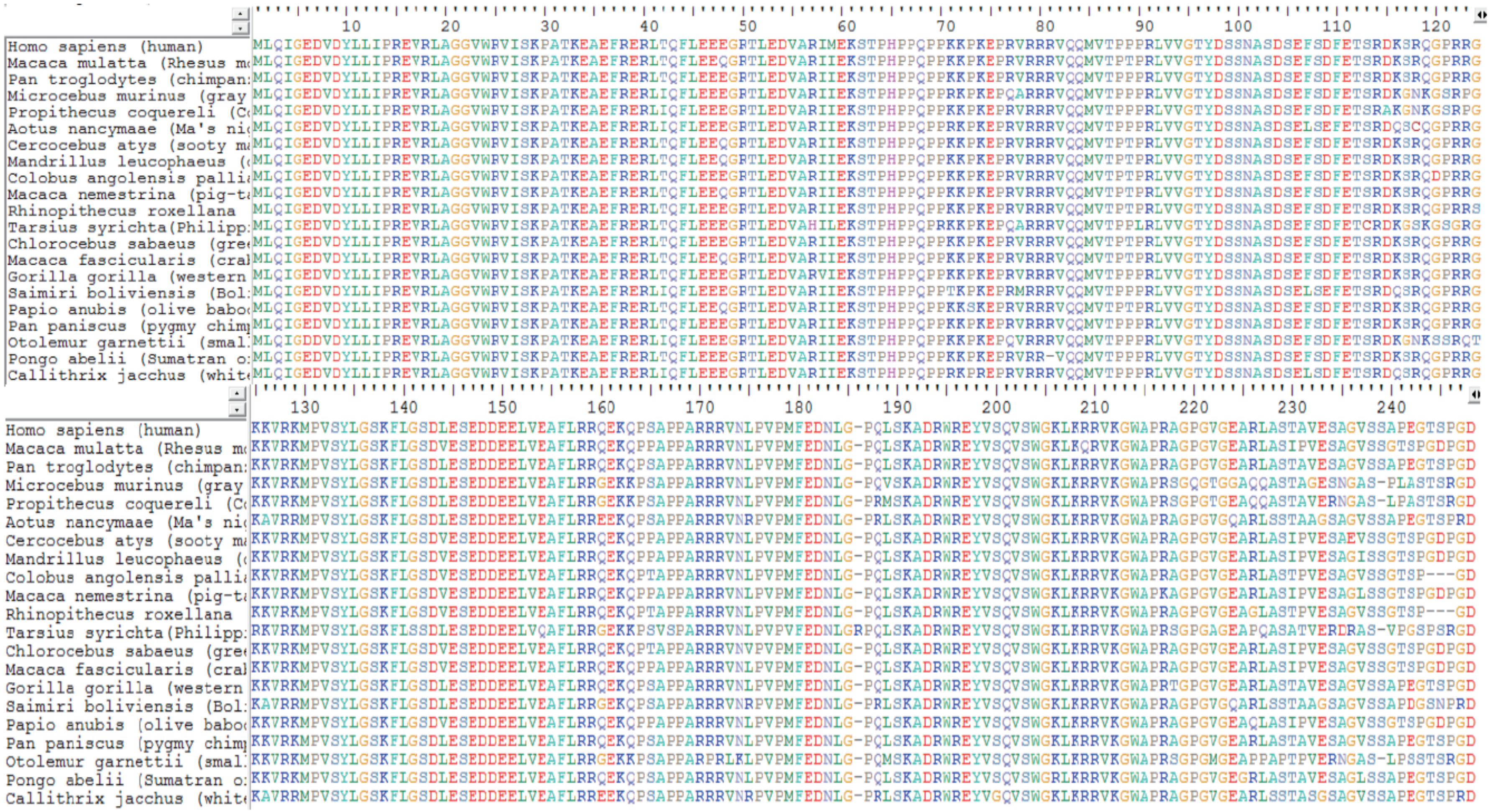

Supplementary Figure S2. Full amino acid sequences of primate CCDC8.

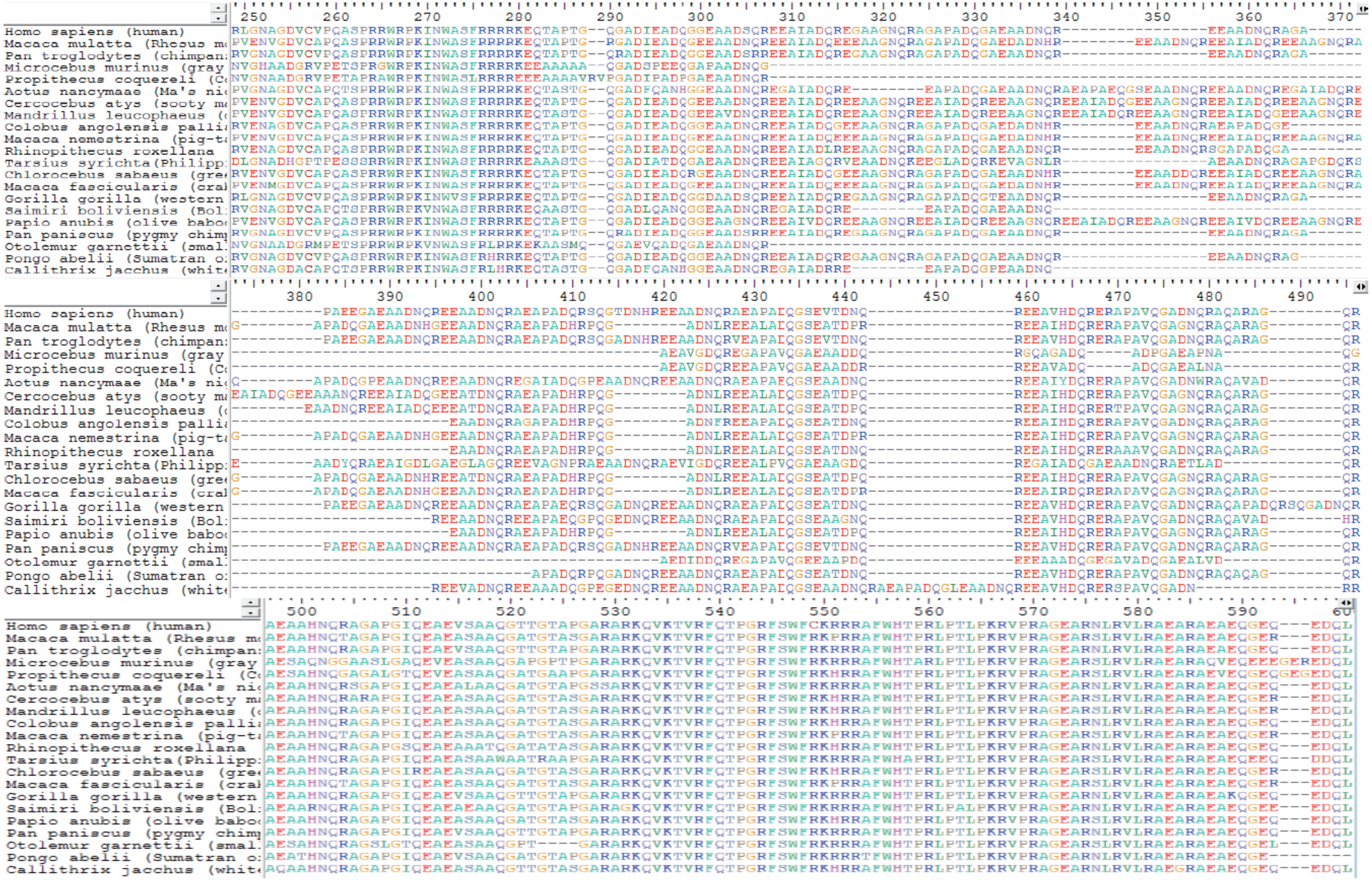

Supplementary Figure S2. Full amino acid sequences of primate CCDC8.

Table S1. Primers for truncated CCDC8 construction

Name	Primer
mCherry-EcoR1-F	5'GGTGGAATTCGTGGGGATGCTGCAGATCGGGGAG3'
mCherry-EcoR1-R	5'CCATGCGGCCGCCAGCTGGTCTTCTTGCTC3'
514R	5'ATATGCGGCCGCTCCTGCCCTGGGGACTCTCTT3'
387R	5'CCATGCGGCCGCCTCTTCCCTGTAGTTATCTGT3'
280R	5'ATATGCGGCCGCCTTCCTGCGGCGCCGAAAGGA3'
279F	5'AATTGAATTCGTGGGGATGGAGCAGACAGCACCC3'
366F	5'ATATGAATTCGTGGGGATGCAGAGGGCAGAGGCC3'
513F	5'ATATGAATTCGTGGGGATGGAGGCCAGGAACCTC3'

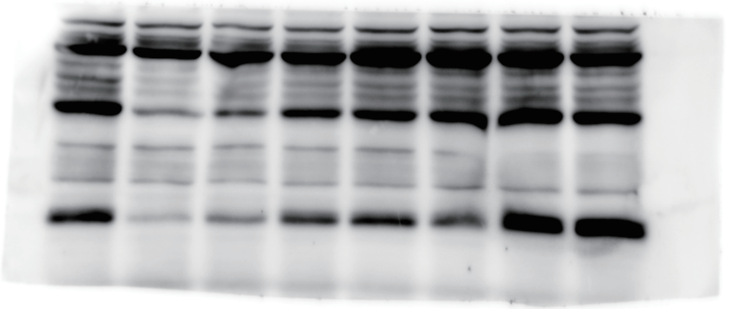

Figure 2D-1

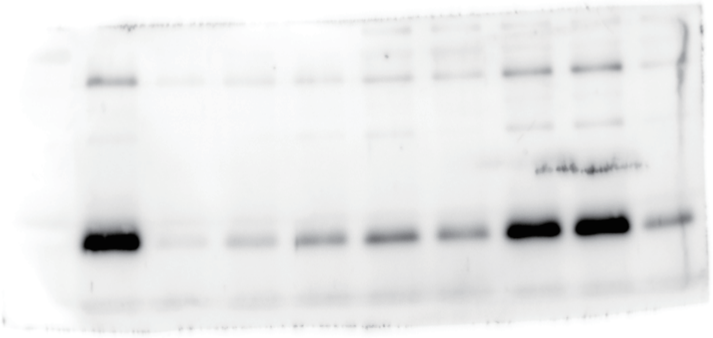

Figure 2D-2

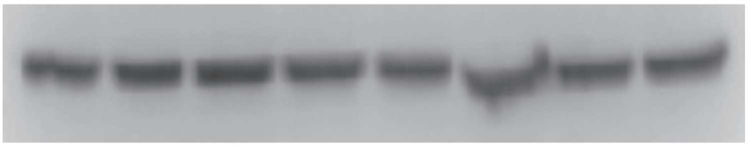

Figure 2D-3

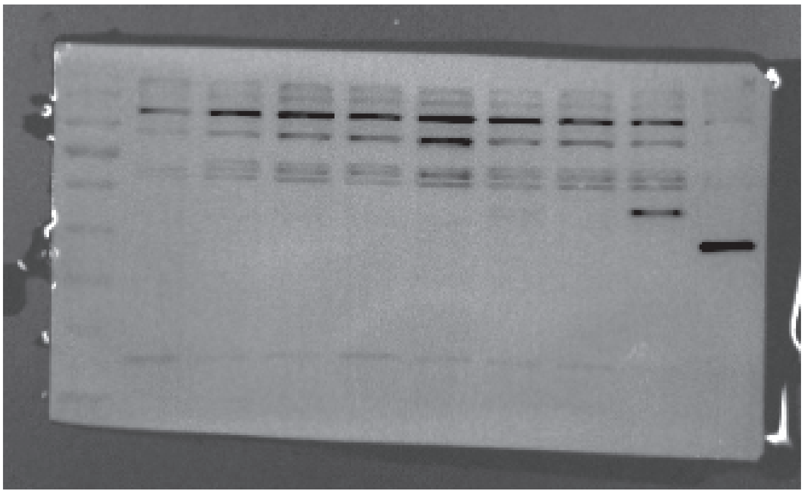

Figure 2D-5

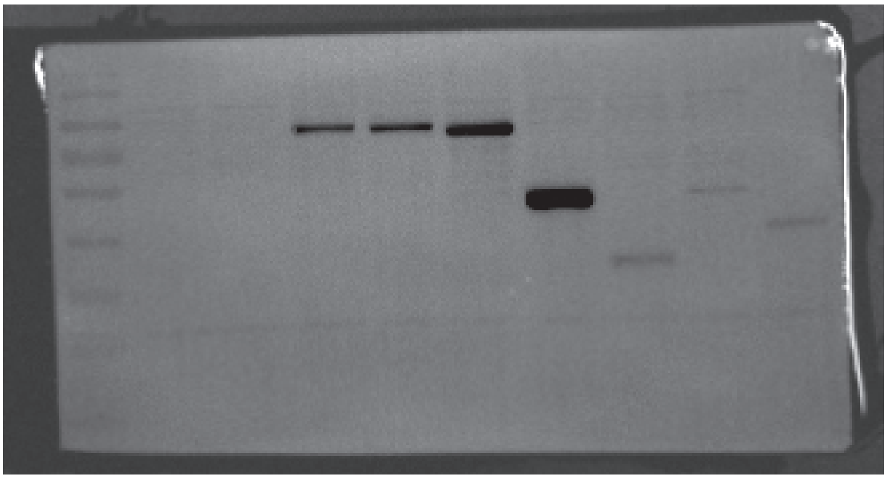

Figure 2D-4

Supplementary Figure S3

These are the original unprocessed Western blot pictures, corresponding to Figure 2D in the article.

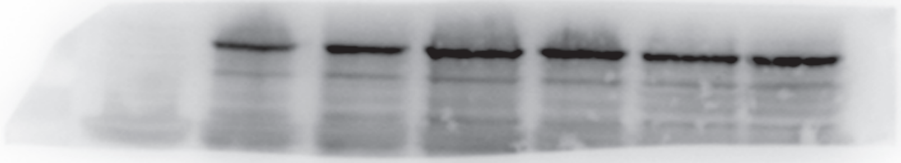

Figure 5D-1

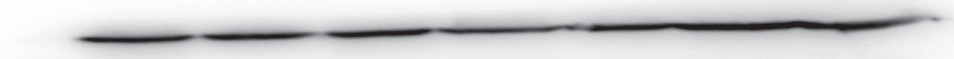

Figure 5D-2

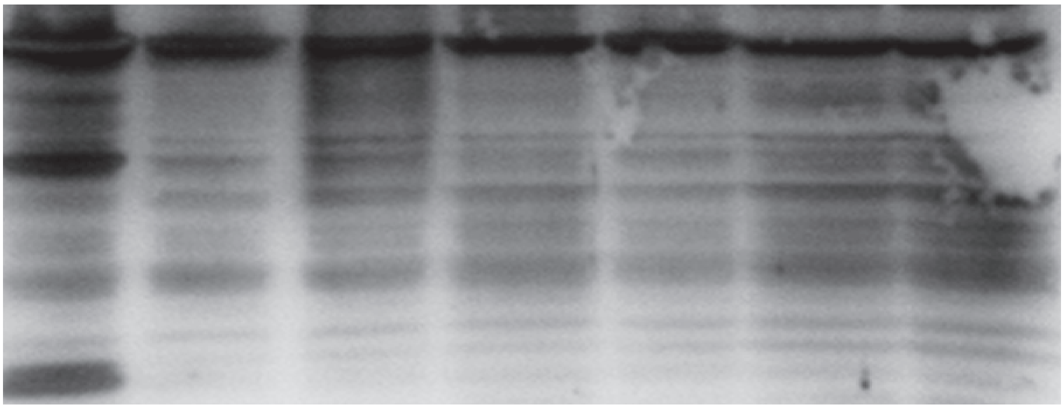

Figure 5D-3

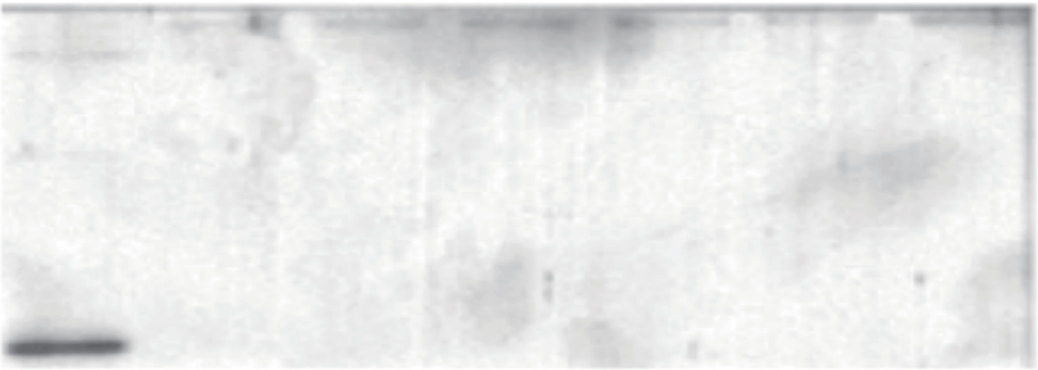

Figure 5D-4

Supplementary Figure S4

These are the original unprocessed Western blot pictures, corresponding to Figure 5D in the article.
